# Molecular aspects of development and regulation of endometriosis

**DOI:** 10.1186/1477-7827-12-50

**Published:** 2014-06-13

**Authors:** Yana B Aznaurova, Marat B Zhumataev, Tiffany K Roberts, Alexander M Aliper, Alex A Zhavoronkov

**Affiliations:** 1 I.M. Sechenov First Moscow State Medical University, Moscow, Russian Federation; 2 Department of Pathology and Laboratory Medicine, Emory University, Atlanta, GA, USA; 3 The First Open Institute for Regenerative Medicine for Young Scientists, Moscow, Russian Federation; 4 The Biogerontology Research Foundation, London, UK; 5 Federal Research and Clinical Center for Pediatric Hematology, Oncology and Hematology, Moscow, Russian Federation; 6 Moscow Institute of Physics and Technology, Moscow, Russian Federation

**Keywords:** Endometriosis, Reproductive age, Bioinformatics analysis, Signaling pathways, Biomarkers, Therapeutic targets

## Abstract

Endometriosis is a common and painful condition affecting women of reproductive age. While the underlying pathophysiology is still largely unknown, much advancement has been made in understanding the progression of the disease. In recent years, a great deal of research has focused on non-invasive diagnostic tools, such as biomarkers, as well as identification of potential therapeutic targets. In this article, we will review the etiology and cellular mechanisms associated with endometriosis as well as the current diagnostic tools and therapies. We will then discuss the more recent genomic and proteomic studies and how these data may guide development of novel diagnostics and therapeutics. The current diagnostic tools are invasive and current therapies primarily treat the symptoms of endometriosis. Optimally, the advancement of “-omic” data will facilitate the development of non-invasive diagnostic biomarkers as well as therapeutics that target the pathophysiology of the disease and halt, or even reverse, progression. However, the amount of data generated by these types of studies is vast and bioinformatics analysis, such as we present here, will be critical to identification of appropriate targets for further study.

## Background

Endometriosis is considered to be a benign gynecological condition. However, it is extremely common, affecting up to 10% of women of reproductive age and can present with severe symptoms such as chronic pelvic pain, pain with menstruation, pain during intercourse (dyspareunia), and dysmenorrhea [1]. While the underlying pathophysiology of endometriosis remains elusive, it is known to be a hormone-dependent disease characterized by ectopic endometriotic lesions. In fact, the pain associated with the severe deep-infiltrating form of the disease is frequently due to the penetration of the muscularis propria by endometriotic lesions in the peritoneal cavity, fallopian tubes, rectosigmoid colon, and bladder [2]. Furthermore, endometriotic lesions can result in scarring of the reproductive organs causing infertility in up to 30% of women who suffer from endometriosis [3,4]. There are many theories regarding the pathogenesis of endometriosis and extensive analysis of the involved signaling pathways will lay a critical foundation for the development new therapies.

### Epidemiology: the rise of endometriosis

The exact prevalence of endometriosis in the population today is difficult to determine because it is asymptomatic or subclinical in the majority of cases. The prevalence has been estimated to be as low as 1% among asymptomatic women and as high as 60% in women with chronic pelvic pain [5,6].

A 2012 study of a German insurance cohort found that the overall prevalence was approximately 0.8% [5]. However, this particular study was enriched for asymptomatic patients in that four age matched controls were identified for each symptomatic patient. This is contrary to the vast majority of studies which are enriched for symptomatic patients, such as a 2013 study in which 62% of adolescent girls being followed for either chronic pelvic pain or dysmenorrhea were diagnosed with endometriosis [6]. Such disparate reports emphasize the complexity of determining, with any certainty, either the prevalence or incidence of this disease.

While some of the more recent studies have suggested that the prevalence of endometriosis may actually be lower than was previously thought, there has been a perceived increase in the incidence of endometriosis in the past several decades [5,7]. This perception may be due to a combination of increased awareness and better diagnosis.

### Risk factors

#### Age

As a hormone-dependent disease of continued endometrial growth, endometriosis is predominantly a disease of women of reproductive age. Endometriosis is rarely found in pre-adolescents with an incidence of only 0.05% in symptomatic patients. In women of reproductive age, the highest incidence was found in women aged 35–44 (0.4%) (relative risk (RR) = 6.3), regardless of symptoms [5]. This is in comparison to women aged 25–34 (RR – 4.0) and 45–54 (RR - 4.5), respectively [5]. However, endometriosis is also frequently diagnosed in symptomatic post-menopausal women (incidence of 2.55%) [8]. It is possible that an increase in endometriosis can be attributed, in part, to the aging of the global population.

#### Lifestyle

• Reduced pregnancy rate

The theory that drastically decreased pregnancy rates as a direct result of the modern woman’s lifestyle, including the availability of contraceptives, contributes to an increased incidence of endometriosis is well established [9-11]. In the absence of frequent pregnancy, the endometrium continually builds-up and then sheds under the control of hormonal signaling. It is thought that the cycling of the endometrium with more frequent menses can lead to endometriosis. Evidence that symptoms of endometriosis resolve during pregnancy has led to frequent treatment with the use of oral contraceptive pills (OCPs) to induce “pseudopregnancy” [12,13]. Even though most women still experience monthly menses while taking OCPs, the hormonal levels simulate pregnancy thereby inhibiting ovulation but also decreasing endometrial build-up and menstrual flow as well as improving endometriosis symptoms.

• Shift work

Menstrual cycle and endometriosis are both driven by circulating estrogen levels [14]. There is evidence to suggest that estrogen levels in women of reproductive age follow a circadian rhythm in addition to the monthly ovarian rhythm and, therefore, may be vulnerable to circadian disturbance [15,16]. In fact, night shift work has been shown to affect estrogen secretion and has been associated with menstrual disruption as well as increased risk of endometriosis [17,18]. One study found that any night shift work at all increased the risk of endometriosis by 50% [14].

#### Toxins

2,3,7,8-Tetrachlorodibenzo-*p*-dioxin (TCDD) and structurally related polyhalogenated aromatic hydrocarbon chemicals (PHAHs) are common environmental contaminants found globally. TCDD and dioxin-like PHAHs are classified as “dioxins” and are associated with a spectrum of toxic effects on the reproductive, immune, and endocrine systems [19]. Additionally, polychlorinated biphenyls (PCBs) contribute to the toxicity of PHAHs including disruption of estrogen activity [20,21].

Dioxins are released into the air as a product of various industrial processes and, therefore, exposure has steadily increased over the last century [22]. The compounds accumulate in the environment and people are exposed through the consumption of animal products as the compounds bioaccumulate in fatty tissues [23]. Food sources account for 93% of dioxin exposure levels in the US population, although inhalation, soil, and water sources may also play a role [24].

A clear relationship between dioxin exposure and the development of endometriosis has been well established in both rodent and primate models [25-29]. However, a causal relationship between the two has not yet been verified in humans though there is evidence to support it. For example, development of ectopic lesions is induced by dioxin exposure in a rodent model using human endometrium [30,31]. Moreover, studies from around the globe have linked an increased risk of endometriosis with elevated concentrations of dioxin in patient serum [32-35].

#### Autoimmunity

Endometriosis is understood to be an inflammatory disease process, which suggests a role for the immune system [36]. It has been postulated that one mechanism for the development of ectopic endometrial lesions is a defective immune response, which fails to clear the implants from the peritoneal surface [36,37]. In fact, immune deficits fulfilling most of the basic criteria for autoimmune disease have been described in endometriosis, including polyclonal B-cell activation, abnormalities in T- and B-cell function, tissue damage, and multi-organ involvement [38,39]. Furthermore, allergies, hypothyroidism, inflammatory bowel disease, and fibromyalgia are among a number of autoimmune conditions known to be associated with endometriosis [40,41]. However; it remains unclear whether these comorbidities may be a cause or an effect of the disease. Perhaps the most convincing evidence that endometriosis may have an autoimmune component is the presence of circulating antibodies towards ovarian and endometrial antigens [42-45]. These auto-antibodies are likely to play a role in both the pathogenesis of endometriosis as well as pregnancy loss in patients with the disease, but further study is needed to firmly establish these links [43,44].

### Etiology

#### Retrograde menstruation theory

Approximately 75-90% of women experience some retrograde intra-abdominal bleeding during menses [46]. The development of endometriosis has been linked to exposure of the pelvic peritoneum to the blood products and cellular debris contained within menstrual fluids, which would normally be confined to the pelvis [46,47]. Furthermore, menstrual fluid also contains some abnormal stem cells, which have been shown to have increased implantation and angiogenic capabilities and can form ectopic tissue lesions in animal models [47,48].

#### Coelomic metaplasia

While retrograde menstruation is the most widely accepted mechanism, it cannot explain rare cases of endometriosis in the absence of a functioning uterus. The coelomic metaplasia theory proposes that endometriosis develops as a result of transformation of mesothelial cells on the ovary to endometriotic gland cells [49,50]. In fact, mesothelial inclusions have been found to be associated with endometriosis in the ovaries, fallopian tube, and pelvic wall [51]. Rare cases of endometriosis described among men, pubertal and adolescent girls, and distant endometriosis in the thoracic cavity support this particular theory [52-54]. Furthermore, an *in vitro* experimental model of human endometriosis demonstrated that ectopic lesions can result from metaplasia of the ovarian surface epithelium [55].

#### Lymphovascular metastasis

The theory of lymphatic and hematogenous spread has long been considered to explain remote occurrence of the disease as well. According to this theory, exfoliated endometrial cells are swept into the venous drainage of the uterus, with subsequent deposition possible anywhere in the body. The theory is supported by the presence of endometriosis in the thoracic cavity and other distant sites outside pelvis as well as detection of endometrial tissue in the uterine vessels in patients with adenomyosis [56]. Lymphovascular metastasis remains a speculative explanation and, while possibly occurring during the development of endometriosis, is not likely to be the primary mechanism as cases of pulmonary and thoracic endometriosis are rare [57,58].

#### Embryonic rest theory

During embryogenesis, some endometrial cells that should grow in the uterus develop in the abdomen instead [59]. These cells would then be activated in puberty under the effects of estrogen and progesterone. Embryogenesis is controlled and directed by a sophisticated, but still incompletely understood, fetal system. This fetal developmental control system may be the fetal analog of the adult immune system. Abnormalities of the fetal development control system may be preserved into adult life, giving rise to detectable abnormalities of the adult immune system [60,61]. The degree of residual abnormality of the adult immune system may control the aggressiveness of the endometriosis that develops, with the result that some patients may develop invasive disease or adhesions, while most do not.

#### Smooth muscle cells

Endometrial stromal cells (ESCs) are the most prevalent cell type in endometriotic lesions. However, smooth muscle cells (SMCs) are also frequently found and have been reported in peritoneal, ovarian, and deep-infiltrating endometriosis [62-65]. Peritoneal SMCs express oxytocin receptors (OTRs), estrogen receptors (ERs), and progesterone receptors (PRs), which are required components of uterine myometrial cells [66]. In contrast, the ability of SMCs to produce contractions has not been demonstrated. It is plausible that peritoneal SM contractions could stimulate peritoneal nociceptors leading to the generation of endometriosis-associated pain [67]. However, whether these SMCs are derived from basal stem cells or reactivated coelomic epithelial cells is still unclear [68].

#### Altered immune response

Macrophages are an integral component of the mononuclear phagocyte system (MPS). They are derived from bone marrow progenitors that enter the circulation as monocytes. After reaching peripheral tissues, they reside as macrophages or antigen-presenting cells, including dendritic cells (DCs). The MPS performs both pathogen eliminating and homeostasis support functions [69,70]. In mouse models, in the absence of macrophages, endometriotic tissue retains the ability to adhere to the peritoneal layer [71]. However, the angiogenic properties were inhibited and endometriotic lesions failed to grow. Infiltrating macrophages have been reported as a consistent feature of endometriotic lesion development in humans. Independent studies have indicated that they are activated by sequence of signals generated within ectopic endometrial lesions or because of the lack of anti-inflammatory hormone-regulated signals in ectopic sites [72-76]. Macrophages are also known to be the source of several chemokines that are involved in endometriosis [71]. However distinct molecular mechanisms that will be useful for diagnostics and treatment remain to be defined.

#### Cellular mechanisms

Endometriosis has long been understood to be a disease of uncontrolled and aberrant growth of endometrial tissue. However, the cellular and molecular mechanisms that are disrupted in this disease remain ill defined. The cell signaling pathways involved can be divided into those involved in proliferation and apoptosis, adhesion and invasion, angiogenesis, and immune function.

#### Proliferation and apoptosis

The mechanisms regulating endometrial cell proliferation are primarily controlled by interactions between the sex steroids and their receptors [77]. Cyclical regulation of cellular proliferation by sex hormones is lost in endometriotic tissue. It is well-known, that alterations in cell cycle molecules such as cyclin and cyclin-dependent kinases are hormone dependent [78]. For example, FOXO1A, a transcription factor involved in cell cycle control and apoptosis, is regulated by progesterone and its expression is significantly reduced in the endometrial tissue of women with endometriosis. Another cell cycle regulatory protein, ErbB-2 (TOB1) is also known to be down-regulated in women with endometriosis, which may be the result of increased interleukin (IL)-1β levels.

**Figure 1:**
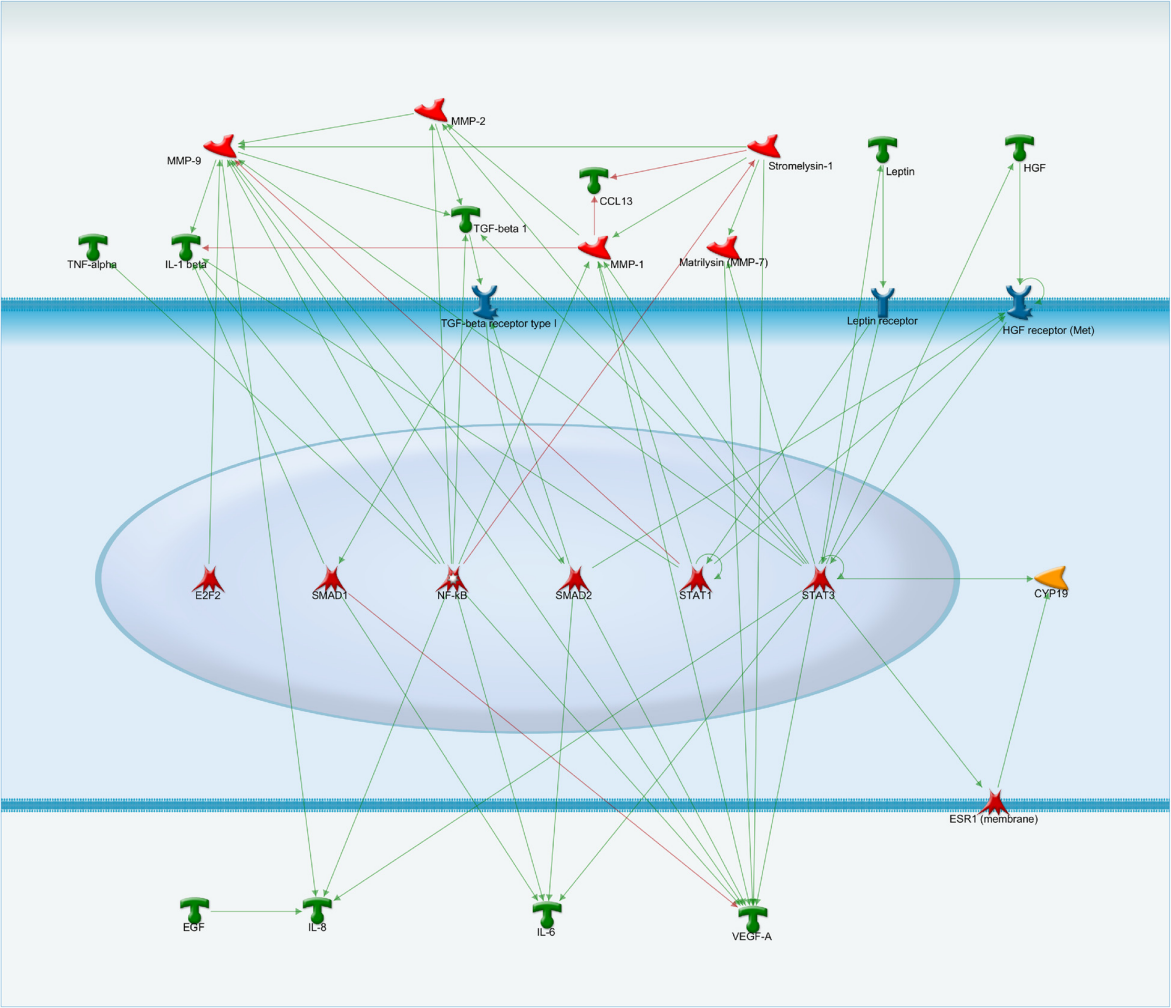
**Transcriptional regulation of proliferation’s factors.** NF-kB, STAT and SMADs are the main regulators of such factors as MMP-2, MMP-9, MMP-7, leptin, HGF, TNF-alpha, which can stimulate proliferation of endometriotic lesions.

Growth factors also contribute to the increased proliferative potential of cells derived from endometriotic lesions. In fact, epidermal growth factor (EGF) is confirmed to stimulate proliferative activity in these cells [79,80]. Mitogen inducible gene 6 (MIG6) is a negative regulator of EGF signaling. MIG6 is down-regulated in women with endometriosis and may, therefore, contribute to unmitigated growth of endometrial cells. Midkine (MK) is a member of the heparin-binding growth factor family that is over-expressed in the ectopic endometrium, which has been implicated in proliferation, migration, angiogenesis, and fibrinolysis [81] [Figure 1].

The recurrent bleeding that is a hallmark feature of endometriosis leads to continual thrombin generation, which can subsequently stimulate proliferation of endometriotic cells via protease-activated receptor 1 (PAR1) [77]. PAR1 downstream signaling induces expression of monocyte chemoattractant protein-1 (MCP1), tissue necrosis factor alpha (TNFα), interleukins (IL), cyclooxygenase-2 (COX-2), matrix metalloproteinases (MMP), hepatocyte growth factor (HGF), and tissue factor (TF) [77]. Inhibition of COX-2 effectively reduces endometriotic epithelial cell proliferation [82]. Furthermore, TNFα, various interleukins, and HGF, which are known to be significantly elevated in the peritoneal fluid of women with endometriosis, also contribute to proliferation of endometriotic cells [83,84].

Leptin is primarily known as the protein released by fat cells. However, leptin is also found at elevated levels in the peritoneal fluid and serum of patients with endometriosis [85]. Leptin expression can be stimulated by pro-inflammatory cytokines such as TNFα and IL-1 and can, in turn, stimulate proliferation of ectopic endometriotic cells [77,85]. Retinoic acid (Vitamin A), on the other hand, is known to protect against endometriosis [86]. However, the retinoic acid catabolic enzyme CYP26A1, is a progesterone responsive gene that is upregulated in women with endometriosis [87]. By inactivating retinoic acid, CYP26A1 contributes to establishment of endometriotic lesions.

The Aryl Hydrocarbon Receptor (AHR) transcription factor is recognized as the culprit for most toxic responses observed after exposure to PAH (Polycyclic aromatic hydrocarbons), dioxins, and PCBs. AHR can affect cellular signaling through interactions with various regulatory and signaling proteins, including those mediated by the estrogen receptor (ESR) and NF-κB (nuclear factor of kappa light polypeptide gene enhancer in B cells) [88]. AHR activation leads to decreases in both the number of ESRs and ESR responsiveness, as well as increases in ESR metabolism. Activated AHR complexes associate directly with ESR-α and -β in the absence of estrogen resulting in transcriptional activation of canonically estrogen-dependent genes. By contrast, in the presence of estrogen, ligand-bound AHR exhibits anti-estrogenic effects by suppressing estrogen-bound ESR-mediated DNA binding. AHR may also be involved in cell-cycle regulation through growth factor signaling, cell-cycle arrest, and apoptosis.

In addition to increased proliferation, the cells that comprise endometriotic lesions are thought to have defects in apoptotic signaling pathways. AHR also interacts with nuclear factor kappa-B (NF-κB) signaling pathways [89-91]. The pleiotropic transcription factor, NF-κB has been identified to protect cells from apoptosis. The protein is constitutively active in endometriotic cells and its activation by lipopolysaccharide (LPS) can induce proliferation of endometriotic cells [92]. B-cell lymphoma/leukemia-2 (Bcl-2) is a well-known anti-apoptotic signaling protein. In normal endometrium, Bcl-2 demonstrates cyclical expression decreasing during the menstrual and late proliferative phases, indicating hormonal regulation. However, this regulation is lost in endometriosis [77]. Conversely, expression of the pro-apoptotic protein Fas is unchanged while its ligand, FasL, is upregulated in endometriotic tissue as well as the peritoneal fluid of women with endometriosis [93,94]. There is evidence to suggest that macrophage derived growth factors, including platelet-derived growth factor and transforming growth factor beta (TGF-β), may stimulate Fas mediated apoptosis of immune cells, which may contribute to an immune-privileged environment for endometriotic cell survival [94]. Furthermore, upregulated expression of survivin, decreased terminal effector caspases and DNA fragmentation factor 45 in endometriotic tissues may be a reflection of resistance against apoptosis at ectopic sites [77].

Watanabe et al. demonstrated that survivin plays a critical role in susceptibility of endometrial stromal cells (ESCs) to apoptosis [95]. Survivin treatment of ESCs leads to a reduction of apoptosis inhibiting proteins, such as cIAP-1, XIAP, and survivin as well as an increase of apoptotic cells [95].

ESCs have been shown to be resistant to IFN- γ treatment, which inhibits the proliferation and apoptosis of EuSCs and NESC. Although the precise mechanism of IFN- γ resistance is unknown, the presence of IFN- γ receptor in ECSC suggests that there is dysregulation of subsequent intracellular signaling pathways in these cells [96].

#### Adhesion and invasion

In order for endometriotic lesions to occur, the cells must invade and implant in distant locations. Increasingly, studies are noting roles for adhesions molecules and growth factors in this process. Cells derived from endometriotic lesions have increased adhesive capacity to various components of the extra cellular matrix (ECM) including collagen type IV, laminin, vitronectin, and fibronectin, whereas normal endometrium is more specific [77,97]. In fact, in the early stages of endometriosis, attachment seems to be due to ECM degradation that could play a key role in initiation of endometriosis [98].

Integrins are a family of cell-cell adhesion molecules that promote cell attachment to the ECM proteins thereby sustaining cell migration and invasion [99]. The β-1 integrins and E-cadherin are both found in the endometrium [99]. Aberrant expression of E-cadherin, β-catenin, and integrins has been reported in endometriosis. β-catenin plays a role in cell-to-cell adhesion and intracellular signaling binding to intracellular E-cadherin and connecting E-cadherin to the cytoskeleton of the cell [100]. The E-cadherin–β-catenin complex plays a crucial role in epithelial cell–cell adhesion and in the maintenance of tissue architecture [101]. Aberrant expression of cadherins and integrins is involved in initiation and progression of human tumors [102]. In the case of endometriosis, there are controversial reports about expression levels of these adhesion proteins.

Poncelet et al. have reported reduced expression of E-cadherin in ESCs [103]. Loss of E-cadherin expression may be related to the local aggressiveness and invasiveness of peritoneal endometriotic lesions [101]. Indeed, Gaetje et al. found in an *in vitro* study using peritoneal endometriotic biopsies that E-cadherin positive cells were devoid of invasive capacities whereas E-cadherin negative cells were invasive [104]. Conversely, Ueda at al. did not find altered expression of E-cadherins in peritoneal endometriotic lesions compared to eutopic endometrium [102]. This data are supported by other studies, which have also found high E-cadherin expression in endometriotic lesions, with no difference compared to proliferative endometrium [101,105]. E-cadherin expression in endometrial cells has been reported to be constant throughout the menstrual cycle [105,106]. However, another study found that E-cadherin mRNA was significantly lower at the proliferative phase than at the secretory phase [107]. Thus, E-cadherin expression can probably be affected by menstrual cycle phase and stage of endometriosis. Hereby, E-cadherin expression patterns in endometriotic tissues are contradictory and the role of E-cadherin in the development and progression of endometriosis is still unclear.

In recent studies, β-catenin has been shown to be down-regulated in endometrioid carcinoma and reduced β-catenin expression could be involved in the pathogenesis of endometriosis contributing to its invasive character. Others have suggested that increased expression of β-catenin and activation of Wnt/β-catenin complex may be a molecular mechanism of fibrosis in endometriosis [101,108,109].

Interestingly, endometriosis showed decreased β-catenin expression compared with endometrioid carcinoma. This implies that different alterations in the E-cadherin–β-catenin complex contribute to the pathogenesis of endometriosis and endometrioid carcinoma. It seems logical that different changes in epithelial adhesion molecules participate in the initiation and/or disease progression of a benign as opposed to a malignant disease [99].

Wnt/β-catenin complex regulates stem cell pluripotency and cell development, integrating signals from other pathways, such as TGF- β and FGF (Fibroblast growth factor), and targeting genes involved in cell migration and proliferation [110]. In particular, TGF- β has been reported to be involved in the pathogenesis of endometriosis, playing a critical role in migration and proliferation of fibroblasts to develop endometriotic lesions [108].

P-cadherin is the predominant cadherin subtype present in the human peritoneum and P-cadherin mRNA has been found to be significantly increased in peritoneal endometriotic lesions compared with eutopic endometrium, suggesting that P-cadherin may be involved in mediating endometrial–peritoneal cell interactions in the development of endometriosis [111].

Integrins mediate adhesion of cells to ECM components, such as collagen types I and IV, fibronectin, and laminin. Integrins are a large family of transmembrane glycoproteins that have a dimeric structure of α and β subunits and act as receptors for ECM components. There are several studies investigating the aberrant expression of integrins in endometriotic cells and their role in the invasion and attachment of ESCs to different components of the ECM [99]. Integrins β1 and β5 were present in endometriotic lesions in a nude mouse model and shown to be of peritoneal origin [112]. Higher levels integrin of α1, α2, αv, β1, and β3 protein expression were observed in ESCs than in (normal eutopic endometrial cells) NESCs. On the other hand, the levels of integrin α3 and αL proteins were lower in ESCs than in NESCs [61]. Integrin α3β1 is weakly expressed in menstrual endometrium and integrin α6β1is strongly expressed, both have been characterized as the principal laminin receptors. Blockage of the β6 subunit by a specific antibody has led to a significant reduction of adhesion of ESCs to laminin and a smaller reduction to other ECM components [113]. Adhesion to fibronectin is mediated by the α4β1, α5β1 and αvβ3 integrins. Blockage of the β1 subunit and RGD (Arg-Gly-Asp) antibodies that are involved in α5β1 and αvβ3 integrin function have not prevented adhesion of ESCs to fibronectin. Blocking of collagen receptors α1β1, α2β1, α3β1 did not reduce adhesion of menstrual endometrial cells to collagen. These data suggest that α6β1 could play a key role in early phases of the development of the endometriosis [113]. Previous studies investigating the role of integrins in menstrual endometrium attachment have not shown the complete inhibition of adhesion to ECM components that suggest other mechanisms to be involved [113].

Osteopontin (OPN) is a glycoprotein involved in cell adhesion and migration by binding to integrins [114]. OPN levels are increased in both the blood and ectopic endometrium of women with endometriosis [114]. OPN is also speculated to influence migration and angiogenesis by regulating CD133+, also known as prominin-1, progenitor cells [114]. The migration of these progenitor cells is thought to contribute to the establishment of distant endometriotic lesions.

Octamer-binding transcription factor 4 (OCT4) is a pluripotent factor that has been reported to be overexpressed in endometrial lesions [115,116]. The expression of OCT4 may contribute to the pathology of ectopic endometrial growth by stimulating the migration activity of endometrial cells [115].

Matrix Metalloproteinases (MMPs) also contribute to cell migration via break down of ECM components and subsequent tissue remodeling. MMP-1, -2, -3, -7, and -9 are upregulated in endometriosis and their expression is induced by cytokines such as IL-1, IL-8, and TNF-α [117,118]. Furthermore, expression of tissue inhibitor of metalloproteinase-1 (TIMP-1) is decreased in the peritoneal fluid of women with endometriosis [119]. TIMPs have been shown to control endometriotic cell migration induced by MMPs, suggesting that its downregulation is a major factor in the pathophysiology of endometriosis.

#### Angiogenesis

**Figure 2:**
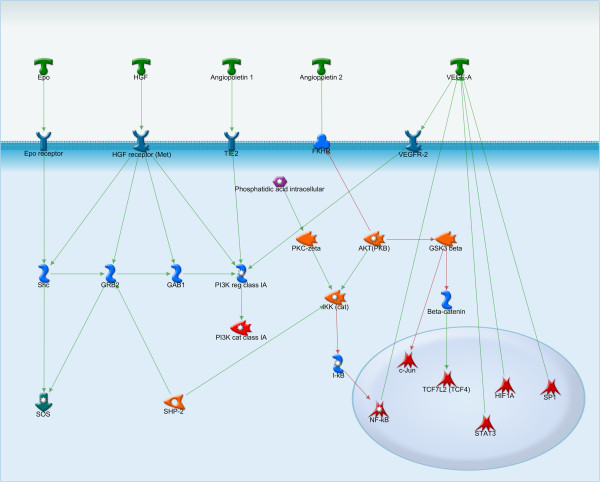
**VEGF and angiopoietin – two main regulators of angiogenesis.** Angiogenesis of lesions is essential for endometriotic cell survival and development. Signaling pathways of VEGF and angiopoietin intersect at PI3K.

Just as observed for tumor growth, angiogenesis of lesions is essential for endometriotic cell survival and development. The two main regulators of angiogenesis are vascular endothelial growth factors (VEGF) and angiopoietins [120] [Figure 2]. VEGF is a key regulator of both physiological and pathological angiogenesis. VEGF is significantly increased in the peripheral blood, peritoneal fluid, and endometrium of patients with endometriosis and its expression is known to be stimulated by a variety of cytokines, including IL-1 [121]. Inhibition of VEGF has been shown to lead to a significant decrease in the number of endometriotic lesions [122]. Angiopoeitin-1 (Ang-1) and Ang-2 are both increased in the endometrium of patients with endometriosis [120,123]. Ang-1 stimulates new vessel formation and Ang-2 can loosen cell-cell and cell-ECM contacts resulting in vessel remodeling.

Glycodelin is an endometrium-derived protein known for its angiogenic, immunosuppressive, and contraceptive effects. Glycodelin is thought to be involved in both the development of endometriosis and the infertility associated with the disease [124].

Glycodelin produced in the glandular epithelium of secretory endometrium and is shed from endometriotic lesions into the peritoneal fluid and serum.

These findings indicate that proangiogenic factors have pivotal roles in the pathogenesis of endometriosis [125,126].

#### Altered immune function

Some theories suggest that the immune system must be at least partially compromised to allow the development of ectopic endometriotic lesions. Macrophages play an especially important role being the predominant leukocytes found in the peritoneal fluid of women with endometriosis [71,127]. They have been shown to be involved in ectopic endometrial cell adhesion, implantation, and growth. Moreover, the secretory products of macrophages are also significantly increased in both the peritoneal fluid of patients with endometriosis as well as in the endometriotic lesions.

One of the major secretory products of macrophages is TGF-β, which is known to play a role in increasing the rate of post-surgical adhesion formation suggesting that it may also play a role in endometriotic cell adhesion [77]. IL-1 is a macrophage derived cytokine that can induce expression of COX-2 and IL-8, thereby likely playing a role in proliferation, migration, as well as angiogenesis of endometriotic lesions [83]. Many of the genes involved in endometriosis are implicated in aging [128]. HGF is also upregulated in endometriotic lesions. HGF can be upregulated by LPS-stimulated macrophages in endometriotic lesions and subsequently enhance proliferation of endometriotic cells [129]. Macrophages are also potential sources of the increased VEGF in patients with endometriosis. Studies in mouse models demonstrate that after implantation of uterine tissues into the peritoneum, macrophages are activated and VEGF is secreted in response to TNF-α and IL-6 [127].

Prostaglandin-E2 (PGE2) is another secretory product of macrophages that is also produced by ectopic endometrial cells [130,131]. PGE2 plays multiple roles in the pathophysiology of endometriosis via signaling through four receptors. Firstly, PGE2 increases estrogen synthesis by up regulating steroidogenic acute regulatory protein (StAR) and aromatase [130]. PGE2 in combination with IL-4 may enhance estrogen production in endometriotic tissues, implying an elaborate mechanism by which the Th2 immune response augments inflammation-dependent progression of the disease [131]. Furthermore, through its effect on estrogen and up regulation of VEGF, PGE2 affects leukocyte populations and promotes angiogenesis. It also inhibits apoptosis and up regulates fibroblast growth factor-9 (FGF-9) to promote cell proliferation.

Lastly, macrophage migration inhibitory factor (MIF) is a cytokine that is a major immune regulator as well as a potent angiogenic and tissue remodeling factor. MIF is significantly increased in endometriotic lesions and is likely upregulated by IL-1 in this context [132].

Natural Killer (NK) cells are a major component of immune surveillance and NK cell activity in the peritoneal fluid can suppress formation of ectopic lesions [133]. Therefore, it follows that their activity is decreased in the peritoneal fluid of women with endometriosis [134]. Soluble intercellular adhesion molecule-1 (sICAM-1), one of the major adhesion molecules that inhibits natural killer cell–mediated cytotoxicity, is involved in the implantation and development of endometriotic lesions [135,136]. Studies have confirmed changes in sICAM-1 levels in women with endometriosis compared with controls [137,138].

Endometriosis is an inflammatory disease associated with abnormal T-cell function. IL-4, a cytokine produced by helper T-cells (Th) is significantly upregulated in endometriotic lesions and can stimulate the proliferation of endometriotic cells [139]. Th17 cells are also enriched in the peritoneal fluid of women with endometriosis as well as the ectopic endometrium. IL-17 has been shown to stimulate IL-8 and COX-2 expression thereby enhancing proliferation and migration of endometriotic cells [140].

Monocyte chemotactic protein-1 (MCP-1) is a member of the small inducible gene family, which plays a role in the recruitment of monocytes to sites of injury and inflammation [141]. Levels of MCP-1 are increased in the peritoneal fluid and serum of women with endometriosis, particularly in patients with early disease [142].

Lastly, mast cells are the major effectors of allergic responses and have been found in increased numbers in ectopic endometrium [77]. Mast cells are likely associated with the fibrosis and adhesion of the lesions. Based on rat models, there is a strong correlation between endometriosis and allergies [77].

Perhaps the most important component of immune dysregulation in endometriosis is mediated by the major histocompatibility complex (MHC). The MHC, also known as Human Leukocyte Antigens (HLA), are cell surface proteins that mediate interactions between immune responsive cells. Aberrant expression of both Class I and II MHC antigens in endometriotic lesions inhibits the cytotoxic activity of NK cells [143,144]. Some studies have suggested that the class I antigens HLA-B*07 and B*46 are associated with the development of endometriosis, whereas HLA-B*48 may offer a protective effect [145,146]. Additionally, the class II HLA-DR antigens are aberrantly expressed in glandular cells of endometrium in endometriosis and adenomyosis and are thought to be involved in various immunological abnormalities [147,148]. Non-classical HLA-G proteins have been suggested to be expressed on ectopic endometriotic cells and to play a critical role in the development of endometriosis though the suppression of NK function [149,150]. However, other studies have reported that HLA-G is not expressed by endometrial cells at all [151].

Despite what is known about altered MHC expression, it is equally plausible that abnormalities in NK receptors could lay the basis of altered immune response in endometriosis. For example, polymorphisms in killer cell immunoglobulin-like receptors (KIRs) may be associated with susceptibility for endometriosis [152].

#### MicroRNA

**Figure 3:**
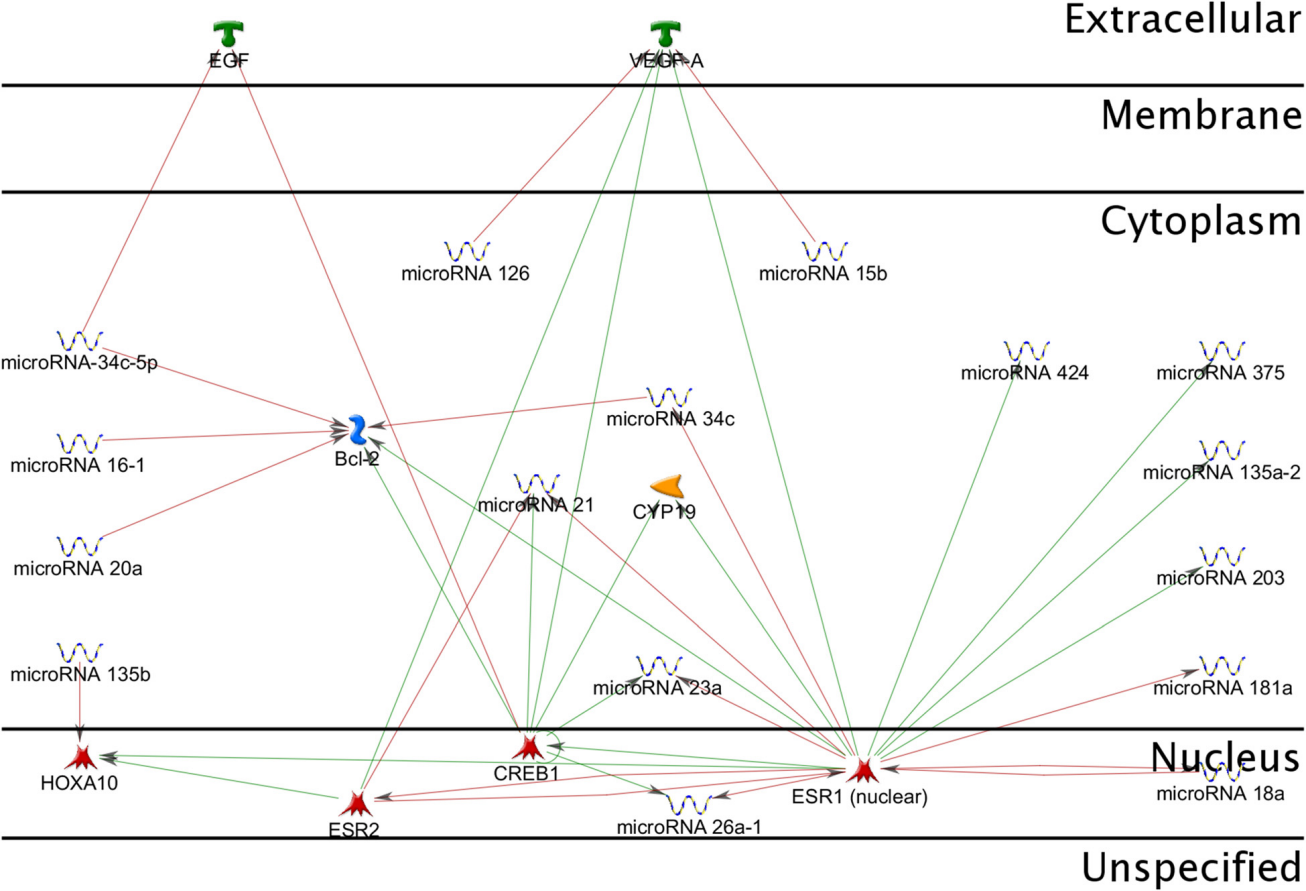
**MicroRNA involved in endometriosis.** MicroRNAs (miRNAs) are naturally occurring posttranscriptional regulatory molecules that potentially play a role in endometriotic lesion development. A number of miRNAs are regulated by 17β-estradiol in the endometrial epithelial and stromal cells, including miR-21, miR-23a, miR-26a-1, miR-203, miR-181a, miR-424, and miR-34c. MicroRNA-15b, miR-126 have been predicted to target the expression of VEGF-A; miR-34c-5p – the expression of EGF.

MicroRNAs (miRNAs) are naturally occurring posttranscriptional regulatory molecules that potentially play a role in endometriotic lesion development [Figure 3]. In one study, 22 endometriosis-associated miRNAs were identified by microarray analysis in paired ectopic and eutopic endometrial tissues [153]. Of these, 14 were found to be up-regulated (miR-145, miR-143, miR-99a, miR-99b, miR-126, miR-100, miR-125b, miR-150, miR-125a, miR-223, miR-194, miR-365, miR-29c and miR-1) and 8 down-regulated (miR-200a, miR-141, miR-200b, miR-142-3p, miR-424, miR-34c, miR-20a and miR-196b) miRNAs [153]. Functional analysis indicated that 673 miRNA targets constitute molecular pathways involved in the development of endometriosis, including c-Jun, CREB-binding protein, protein kinase B (Akt), and cyclin D1 (CCND1) signaling [141]. Another study found 10 microRNAs that were up-regulated (miR-202, 193a-3p, 29c, 708, 509-3-5p, 574-3p, 193a-5p, 485-3p, 100, and 720) and 12 that were down-regulated (miR-504, 141, 429, 203, 10a, 200b, 873, 200c, 200a, 449b, 375, and 34c-5p) in endometriosis compared with normal endometrium [154].

A number of miRNAs are regulated by 17β estradiol and progesterone in the endometrial epithelial and stromal cells, including miR-20a, miR-21, miR-23, miR-26a, miR-18a, miR-181a, miR-206, and miR-142-5p. These miRNAs have been predicted to target the expression of a large number of genes, including transforming growth factor β (TGF-β), TGF-β receptors, ERs, PRs, and CYP-19A1 (aromatase), many of which are known to play critical roles in endometrial activities [155].

Additional studies have explored individual miRNAs in eutopic endometrium from women with endometriosis. Compared with healthy controls, endometrium from women with endometriosis is characterized by over-expression of miR-135a in the proliferative phase and miR-135b in the proliferative and secretory phases [156]. These miRNAs were predicted and validated to target HOXA10, a key mediator of endometrial receptivity, the expression of which was simultaneously repressed in the endometrium of women with endometriosis [157]. Transfection of ESCs with miR-135a/b or miR-135a/b inhibitors resulted in altered expression of HOXA10 mRNA and protein and this may suppress endometrial receptivity in endometriosis [157].

Transfection of ESCs with miR-199a repressed IκB kinase/NFκB signaling and inhibited IL-8 secretion. Therefore, the low expression levels of miR-199a in ESCs from women with endometriosis would be expected to up-regulate these inflammatory mediators and result in decreased endometrial receptivity and implantation defects [158].

Decreased miR-20a and miR-200b may contribute to the up-regulation of CREB binding protein (CREBBP) mRNAs in endometriosis [159]. CREBBP is a co-activator of hypoxia inducible transcription factor 1a (HIF1a), a hypoxia induced and pro-angiogenic transcription factor. CREBBP/HIF1a activities are likely to be increased in ectopic endometriotic lesions due to loss of transcript suppression by downregulation of miR-20a and miR-200b. Reduced repression of HIF1a mRNA translation by miR-20a is consistent with the elevated HIF1a mRNA levels seen in endometriotic lesions. CREBBP was also central in one of our miRNA regulated pathway networks associated with angiogenesis in endometriosis.

Both HIFa and NFκB can be activated by elevated levels of IL-1β and TNFα in endometriosis. This process can lead to enhanced COX-2 transcription [160]. COX-2 participates in a positive feed forward loop that enhances aromatase activity and local estradiol production in endometriotic lesions, thereby promoting a proliferative local hormonal environment. COX-2 translation is known to be suppressed by miR-199a and miR-16 and both of these miRNAs were down-regulated in endometriosis [155,161].

Apoptotic resistance is mediated by BCL2, leading to enhanced survival of stressed endometrial cells in endometriosis. BCL2 is targeted by miR-15b/16 and the reduced expression of these miRNAs may contribute to increased activity of this anti-apoptotic protein in endometriosis [162]. Furthermore, cell proliferation is promoted by the cell cycle regulator insulin receptor substrate-1 (IRS1). Two highly up-regulated miRNAs in endometriosis, miR-126 and miR-145, target this mitogenic protein and may inhibit endometrial cell proliferation [153]. The low levels of miR-20a, miR-221 and miR-222 seen in endometriotic tissues may ease post-transcriptional suppression of their mRNA targets, which include the cell cycle repressors cyclin-dependent kinase inhibitors CDKN1A/p21, CDKN1B (p27), and CDKN1C (p57). High miR-15b/16, miR-143, miR-145 and low miR-20a, miR-221 and miR-222 expression are consistent with repressed cell proliferation and enhanced cell survival in endometriosis [163].

Endometriotic lesion development has been associated with an aberrant expression of ECM proteins. Upregulation of miR-29c in endometriotic tissue may control the ECM production that is integral to endometriotic lesion development [153]. Precise organization of the ECM scaffold may be crucial for the correct placement of developing glands and stroma in remodeling tissues and miRNAs may contribute by fine tuning this process.

In endothelial cells, miR-126 enhances VEGF and FGF signaling, leading to neoangiogenesis and the development of mature vasculature [164]. miR-126 is embedded in the EGF-like-domain, multiple 7 (EGFL7) gene and both of these transcripts are highly up-regulated in ectopic versus eutopic endometrium, which is indicative of co-transcription [153,165]. EGFL7 enhances the effect of miR-126 by inducing endothelial cell migration during neovascularization [166].

### Screening and diagnostic techniques

#### Monitoring endometriosis in a clinical setting

Definitive diagnosis of endometriosis is notoriously difficult. Laparoscopy is currently the gold standard for diagnosis [167]. Endometriosis manifests in peritoneal congestion, adhesions, and other defects, which are readily observed by laparoscopy. Additionally, if laparoscopic findings are suspicious a biopsy can be obtained. Histological diagnosis of endometriosis, while confirmative, is often difficult [168]. Indeed, the sensitivity and specificity of either laparoscopy or biopsy are not sufficient to justify routine clinical use either for diagnosis or monitoring. Furthermore, both of these techniques are invasive and, therefore, present a major barrier to effective clinical management of endometriosis.

High-resolution transvaginal ultrasonography and, in particular, MR imaging are increasingly used to diagnose the presence and extent of infiltrating lesions [167]. Transvaginal sonography is useful in the diagnosis of ovarian endometriomata and a pelvic sonogram or ultrasound can detect endometriosis cysts of the ovaries. However, none of these methods are effective to detect endometriotic lesions in the pelvis because endometriosis can look similar to other kinds of ovarian cysts on sonogram. Thus, a surgical evaluation is required if the cyst persists throughout 2 menstrual cycles.

For these reasons, development of sensitive and specific non-invasive tests for endometriosis is a priority for investigators [169]. Many DNA-based non-invasive diagnostics methods are used in the clinical practice in obstetrics and gynecology [170]. A noninvasive test would be useful for early detection and staging of endometriosis in symptomatic women who have pelvic pain and/or subfertility with normal ultrasound results. Currently, there are several diagnostic tests including panels of known peripheral blood biomarkers, protein/peptide markers, and miRNAs. However, no reliable blood test for endometriosis exists [171]. Nonetheless, with the maturation of genomic and proteomic technology we are closer than ever to identifying a blood test for rapid and reliable diagnosis of this debilitating disease. So far, studies have focused on glycoproteins, cytokines, adhesion molecules, as well as angiogenic and growth factors, which are all associated with the pathogenesis of endometriosis and the development of endometriotic lesions [171].

**Figure 4:**
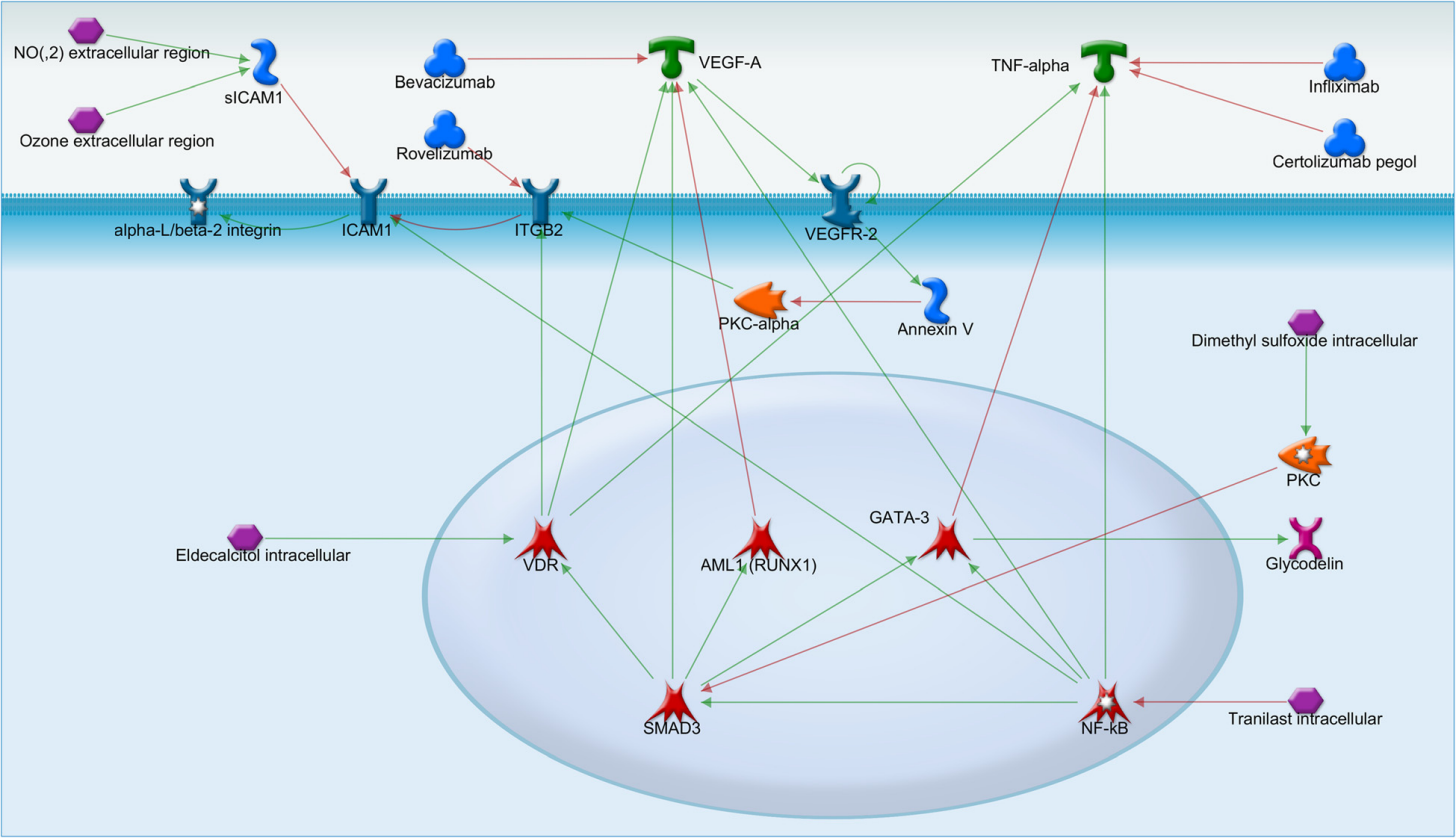
**Intracellular regulation of TNF-alpha, VEGF-A, sICAM1 and annexin V.** VEGF-A may activate annexin V, a marker of apoptosis, through VEGFR-2. TNF-alpha is regulated by such transcriptional factors as VDR, GATA-3, and NF-kB and is involved in signaling pathways of many growth factors. sICAM1, one of the adhesion molecules involved in the implantation and development of endometriotic lesions, is activated by NF-kB. There are a lot of drugs that have an effect on TNF-alpha, annexin V, VEGF-A and might be potentially used in the treatment of endometriosis.

Biomarkers such as annexin V, vascular endothelial growth factor (VEGF), CA-125, and soluble intercellular adhesion molecule-1 (sICAM-1/or glycodelin) [Figure 4] in plasma samples have been shown in the multivariate analysis to diagnose endometriosis that was undetectable by ultrasound with a sensitivity of 81% to 90% and a specificity of 63% to 81% [171]. Specifically, cancer antigen 125 (CA-125) has come into common use as a peripheral biomarker of endometriosis [172]. CA-125 is known to be produced by endometrial and mesothelial cells and enters the circulation in response to inflammation via the endothelial lining of capillaries. Moderate elevation of serum CA125 has been observed in endometriosis, particularly in patients with severe disease [173,174]. However, CA-125 levels in peripheral blood lack diagnostic power as a single biomarker of endometriosis due to low sensitivity [172].

Studies investigating of serum cytokines as biomarkers have demonstrated significant elevation of levels of interleukin-6, monocyte chemotactic protein-1, and interferon-gamma in serum of subjects with endometriosis compared to healthy women. However, the authors suggest that only IL-6 provides a promising serum marker for nonsurgical diagnosis of endometriosis because IL-6 has higher specificity alone then when adding serum IL-6, MCP-1, and INF-g together. Additionally, IL-6 levels did not change during any phase of the menstrual cycle [175]. A study by Wang et al. demonstrated that the circulating miRNAs miR-199a, miR-122, miR-145*, and miR-542-3p could potentially serve as noninvasive biomarkers for endometriosis [146]. This was the first report that circulating miRNAs serve as biomarkers of endometriosis.

One potential method for analyzing the differences between the stages of endometriosis in order to personalize treatment is to apply gene expression analysis and measure the signaling pathway activation profiles [176,177], which could be considered for further investigation of possible personalized science research projects [178]. It is possible that different subsets of biomarkers may be required for the diagnosis of different stages of endometriosis.

#### Epigenetics of endometriosis

The word ‘epigenetics’ refers to the study of heritable changes in gene expression that occur without changes in the DNA sequence [179], which includes mechanisms such as DNA methylation, histone modification, and transcription factor regulation [180]. Cancer and many other diseases show aberrant epigenetic regulation [181]. There is accumulating evidence to support the etiology of endometriosis as an epigenetic disease [182,183]. However, much remains to be studied in order to fully evaluate the role of epigenetic factors in the development of endometriosis.

DNA methylation is the best understood and currently most extensively studied epigenetic mechanism. The modification, specifically the attachment of a methyl group to the 5-carbon position of cytosine bases, occurs within the CpG dinucleotides and is mediated by DNMTs [184,185]. DNA methylation plays an important role in cellular processes and regulation of gene expression and DNA methylation at CpG islands is invariantly associated with gene silencing. The methylated CpGs are docking sites for silencer-type transcription factors that contain a methyl CpG-binding domain (MBD) [186]. DNMTs are divided into two main categories: enzymes involved in the maintenance of DNA methylation (DNMT1) and enzymes involved in de novo DNA methylation (DNMT3A and DNMT3B) [187]. The overexpression of the DNMTs may be a prerequisite for DNA hypermethylation [188]. DNMT1, DNMT3A and DNMT3B are overexpressed in the epithelial component of endometriotic implants. In contrast, only DNMT3A was found to be upregulated in the eutopic endometrium of women with endometriosis [189]. A positive correlation has been noted among these three DNMTs. The upregulated expression of DNMTs in endometriotic tissue suggests that hypermethylation may frequently occur in endometriosis. Several factors have been reported to induce DNA methylation, including aging, diet, chronic inflammation, prolonged transcriptional suppression, and environment [190-193]. These aberrations are likely to be responsible for observed phenotypic aberrations at hormonal, biochemical, inflammatory, immunologic, angiogenic, apoptotic, and, ultimately, clinical levels in endometriosis, which are manifested as the excessive local production of estrogen and prostaglandin, inflammation, development of progesterone resistance, altered apoptotic mechanisms, and implantation failure. Because endometriosis is a persistent disease, it is speculated that chronic inflammation, a feature of endometriotic tissue, may induce aberrant DNA methylation [194]. However, the aging cell undergoes a DNA methylation drift. Early studies showed that global DNA methylation decreases during aging in many tissue types [195]. Several specific regions of the genomic DNA become hypermethylated during aging [196]. Interestingly, genes with increased promoter methylation during aging include the E-cadherin gene, which is downregurated and hypermethylated in endometriotic cells [197,198].

There has been a revolution in DNA methylation analysis technology [194]. A number of aberrantly expressed genes have been reported in endometriosis, which could be related to aberrant DNA methylation [199,200]. Particularly, ERβ up-regulation in endometriotic cells may be related to the hypomethylation of the ERβ-promoter region and decreased PR-B expression in endometriotic tissue could be related with the hypermethylated promoter region of PR-B gene [201,202]. Aromatase is a key molecule for estrogen production and has been demonstrated to be regulated by DNA-methylation in endometriosis [194]. Unmethylated CpG islands within the aromatase gene in endometriotic cells may lead to its up-regulation [194,203]. Downregulation of E-cadherin has been shown in endometriotic cells and may occur due to hypermethylation at the promoter region [198,204]. Steroidogenic factor-1 (SF-1) is a transcriptional factor essential for estrogen biosynthesis and has aberrant expression in endometriotic lesions compared to eutopic endometrium [205-207]. Increased expression of SF-1 in endometriotic cells is related to demethylation of the SF-1 gene promoter that leads to interaction with SF-2, which activates its transcription in endometriotic cells. On other hand, demethylation of the SF-1 gene promoter excludes binding of transcription factor MBD2, which prevents its interaction with transcriptional activators, resulting in silencing of the SF-1 gene [208]. SF-1 expression in endometriosis may enhance aromatase expression leading to local estrogen production [194]. In women with endometriosis, HOXA10 expression is significantly decreased in the eutopic endometrium during the secretory phase, indicating functional defects in uterine receptivity [157,209]. The promoter region of HOXA10 gene was found to be hypermethylated in the eutopic endometrium of women with endometriosis and may be a cause of HOXA10 downregulation [157,210]. Using the epigenetic concept as the lens, new diagnostic markers or therapies may be developed to overcome serious problems in patients with endometriosis.

### Current treatment methods

#### Hormone therapies

Hormone therapy for endometriosis is frequently effective at reducing or even eliminating the pain of the disease [211]. The primary mechanism of action of hormone therapy is to inhibit estrogen production [211].

The success of various hormonal therapies depends on the localization and type of the endometriotic lesions. Superficial peritoneal and ovarian implants seem to respond better to hormone therapy than deep ovarian or peritoneal lesions or lesions within organs [211]. Moreover, hormone treatment has no effect on adhesion of endometriotic cells and cannot improve fertility. Nonetheless, a number of hormonal agents remain the mainstay of endometriosis therapy.

#### Prostaglandin synthetase inhibitors (PGSIs)

PGSIs are a heterogeneous group of non-steroidal inflammatory agents that inhibit the production of prostaglandins [212]. PGSIs are effective in the early stages of endometriosis, but lose efficacy when symptoms become more severe [212]. Nevertheless a lot of side effects such as skin reactions, bronchospasm, and serious blood dyscrasias have been reported for several of these drugs [213].

#### Levonorgestrel intrauterine system

The levonorgestrel intrauterine system (LNG-IUS) is a long-acting contraceptive method, which acts through a steady low level of LNG in the peripheral circulation. The LNG-IUS appears to have a direct effect on the growth of endometriotic deposits through peritoneal fluid [214]. The suppression of menstruation, or marked reduction of flow, may also be beneficial in reducing the amount of retrograde menstruation. One of the side effects of the LNG-IUS is thinning of the endometrium, which causes a decrease in menstrual blood loss and a high incidence of amenorrhea. Thereby the LNG-IUS is used as a treatment for dysmenorrhea, menorrhagia and endometriosis [215].

A study comparing LNG-IUS with expectant management demonstrated significantly lower pain scores in the LNG-IUS participants at 12 months [216]. In the second trial LNG-IUS was compared with a GnRH analogue and found to be equally effective in reducing pain scores after 6 months [217].

#### Oral contraceptives (OCPs)

OCPs contain both estrogen and progesterone and regulate the monthly development of the endometrial lining. Use of OCPs has been suggested to reduce or eliminate the pain associated with endometriosis, making them an attractive long-term treatment option [12]. The most common side effects of OCP treatment are acne, weight gain and irregular withdrawal bleeding [218].

#### Gonadotropin-releasing hormone (GnRH) agonists

GnRH analogues are synthetic hormones that cause an artificial menopause via inhibition of luteinizing hormone (LH) and follicle stimulating hormone (FSH), which in turn decreases estrogen levels preventing menstruation. They can be administered as a nasal spray, by injection, or as an implant [211]. GnRH agonist treatment can force endometriosis into long term remission [219,220]. However, this is at the expense of infertility and other side-effects reminiscent of menopause such as hot flashes, vaginal dryness, and bone loss [211,221]. These side-effects can be minimized by co-administration of a low-dose estrogen or progestin hormone replacement therapy (HRT). Infertility resolves shortly after discontinuation of the medication.

#### Progestogens

Progestogens are synthetic progesterone analogues that prevent ovulation. Both injectable progestogens such as medroxyprogesterone (Depo-Provera) and intrauterine systems (Mirena) have been successfully used to treat endometriosis [211]. The most common side effects are irregular menstrual periods, stopping of menstrual bleeding, weight gain.

#### Antiprogestogens

Also known as synthetic testosterone derivatives, antiprogestogens are synthetic hormones that bring on an artificial menopause by decreasing the production of estrogen and progesterone. Antiprogestogens suppress the growth of the endometrium and the symptoms of endometriosis by blocking the production of ovarian-stimulating hormones (LH and FSH) [211]. Side effects of antiprogestogens comprise acne, weight gain, mood changes and the development of masculine features such as hair growth and a deepening voice.

### Bioinformatics analysis

#### Genome wide association studies (GWAS) – genetic biomarkers

There are four GWAS for endometriosis susceptibility currently listed in the National Human Genome Institute’s catalog [222-225]. Three of which were conducted in a population primarily of Japanese ancestry [222,224,225]. The GWAS listed in the catalog include only those publications attempting to query at least 100,000 single nucleotide polymorphisms (SNPs) in the initial stage. There are also ~20 GWAS that are not listed in the catalog. However, the clinical utility of defining SNPs in endometriosis remains questionable. It is likely that SNP-based testing for endometriosis will convey insufficient diagnostic power and will have be combined with protein biomarkers identified from transcriptome analyses.

#### Transcriptome studies

We analyzed the available microarray data in order to identify differentially regulated signaling pathways that may be involved in the pathogenesis of endometriosis. Further analysis of these pathways may reveal proteins, peptides, or mRNAs that may be useful as biomarkers of the disease or as potential therapeutic targets.

#### Signal transducers and activators of transcription (STATs)

**Figure 5:**
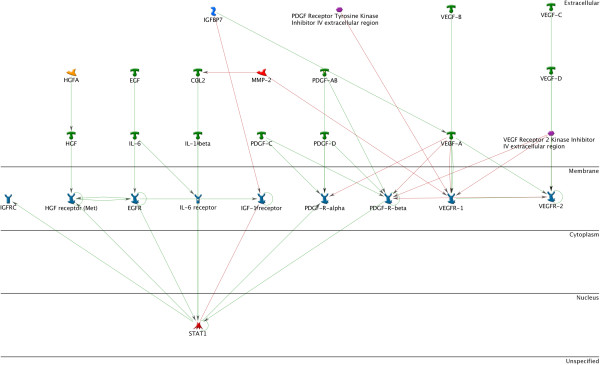
**STAT1 as an integral factor of IGF, HGF, EGF, IL-6, and PDGF signaling pathways.** STAT1, 3, and 5 are primarily activated by growth factors and regulate proliferation and apoptosis. A number of growth factors are known to contribute to the increased proliferative potential of cells derived from endometriotic lesions. The effects of such factors as EGF, PDGF, FGFR, IL-6, HGF and VEGF are all primarily achieved through STAT activation.

Network analysis revealed that signal transducers and activators of transcription (STAT) proteins formed a central node of regulation for a majority of the pathways. STATs are a family of seven transcription factors that reside in the cytoplasm in the inactivated form. The STATs can be divided into two groups based on function. The first group, STAT2, 4, and 6, are activated by cytokines and are involved in T-cell development in IFN-γ signaling [226]. STAT1, 3, and 5 are primarily activated by growth factors and regulate proliferation and apoptosis [226]. A number of growth factors are known to contribute to the increased proliferative potential of cells derived from endometriotic lesions. The effects of EGF, PDGF, FGFR, IL-6, HGF and VEGF are all primarily achieved through STAT activation [Figure 5].

**Figure 6:**
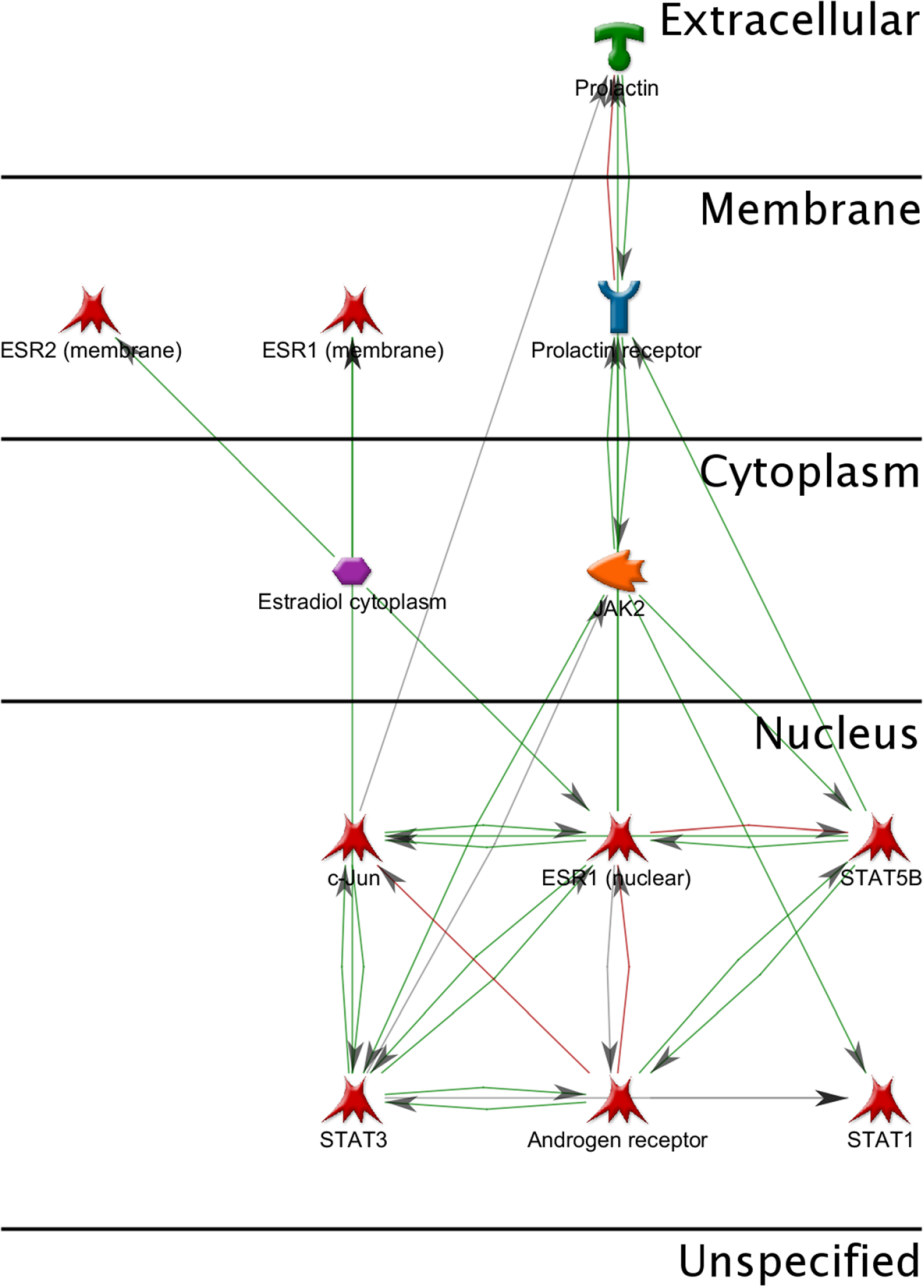
**Intracellular interactions of estradiol, androgens and prolactin.** Estradiol, androgens and prolactin signaling pathways also pass through the STATs. STAT1 and STAT3 contribute to the implementation of their effects.

STATs are latent transcription factors that reside in the cytoplasm. They are primarily activated via C-terminal phosphorylation by Janus kinase (JAK), which induces nuclear translocation of STAT via importin α-5 and the Ran nuclear import pathway [227]. However, some growth factor receptors, including EGFR, HGFR, and PDGFR, have intrinsic tyrosine kinase activity allowing them to directly phosphorylate and activate STAT [228]. Once in the nucleus, dimerized STATs bind specific regulatory sequences to activate or repress the transcription of target genes [227]. STATs mediate effects through transcriptional activation of target genes that enhance proliferation (CCND1 and c-Myc), angiogenesis (VEGFA, ADM and ANGPTL4), invasion (FGA, FGB, CTSB and SERPINE2), and suppression of apoptosis (Bcl-xL, Bcl-2, Mcl-1 and Survivin) [229] [Figure 6].

STAT5 regulates the genetic transcription of cyclins and constitutive activation of STAT5 deregulates the cyclin complexes D/CDK4-6, which control progression from the G1 to the S-phase of the cell cycle [230]. Furthermore, the constitutive activation of STAT5 induces antiapoptotic signals including Bcl-xL [231].

Alternatively, no STAT3 mutations inducing constitutive activation have been identified. Rather, dimerized mutant forms called STAT3C, which require no phosphorylation to become active, are able to migrate to the nucleus, guide transcription, and induce cell transformation [232]. As with STAT5, the constitutive activation of STAT3 both induces proliferation and inhibits apoptosis. STAT3 plays an important role in the G1-S cell-cycle transition since it upregulates cyclins D (D1, 2, 3) and A (cdc25A), and downregulates p21 and p27 [233]. STAT3 can also activate proangiogenic factors such as VEGF [234].

Since endometriosis develops through mechanisms that include proliferation, inhibition of apoptosis, migration, and angiogenesis, it is feasible that STATs may play a key role in the pathogenesis of endometriosis as a master regulator of many of these pathways.

#### SMAD transcription factors

Other key regulators mediating growth factor pathways are the SMAD transcription factors. The SMAD proteins are the only family of transcription factors known to propagate TGF-β signals. However, they also regulate other growth factor pathways, including PDGF, VEGF, and HGF [235].

TGF-β is a serine-threonine kinase that regulates cell proliferation through the phosphorylation of SMADs, which induces their nuclear translocation [236]. Activated TGF-β phosphorylates the R-SMADs (receptor activated), which includes SMAD2 and SMAD3. Phosphorylated R-SMADs bind to SMAD4 and translocate to the nucleus as a complex [236]. SMAD4 is required for the formation of an active transcriptional complex [237].

A diverse array of genes is subsequently regulated by SMAD signaling. Proliferation signals under the transcriptional control of TFG-β and SMADs include cyclin-D1, cyclin-dependent kinase 4, p21, p27, p15, and c-myc [238]. TGF-β is well known to be a pro-apoptotic protein, regulating expression of Bad and caspase-3 [239]. Additionally, ECM proteins including fibronectin, collagen, and MMPs, are also regulated by SMAD signaling [238,239].

In addition to all of these pathways, which are fairly well understood in relation to TGF-β/SMAD signaling, this key regulatory node may also contribute to a compromised immune status, which allows for formation of endometriotic lesions. Firstly, TGF-β secretion from increased numbers of macrophages in the peritoneal fluid of women with endometriosis can induce expression of monocyte chemoattractant protein-1 (MCP-1) as well as COX-2 and PGE2. The upregulation of these signals results in a chronic inflammatory response [239]. Furthermore, SMAD-deficient mice have demonstrated defects in T-cell differentiation leading to an imbalance of effector and regulatory lymphocytes. Loss of immune system homeostasis could contribute to the pathogenesis of endometriosis [240].

#### MEK/ERK

Perhaps the most crucial signaling node identified in our studies is the mitogen-activated protein kinase (MAPK)/extracellular signal-regulated kinase (ERK) or MEK pathway. This pathway has been extensively studied over the past few decades and has well defined roles in regulation of signaling for proliferation, apoptosis, adhesion, invasion, angiogenesis, and evasion of immune surveillance [241,242]. ERK controls transcriptional expression of c-myc and c-Fos, which are both involved in cell-cycle progression and cellular proliferation [243]. In the cytoplasm, ERK is responsible for the regulation of the ribosomal S6 kinase (RSK) family proteins, which subsequently regulate cell-cycle progression via expression of p27, apoptosis via inhibition of Bad, as well a number of other signaling cascades via the transcription factors NF-κB and estrogen receptor-α [243]. ERK also has effects on the ECM via regulation of the actin-binding protein paladin [243]. The contribution of MEK/ERK to cell adhesion, migration, and invasion is partly mediated through its regulation of the down-stream TGF-β/SMAD pathway [242]. Lastly, the MEK/ERK pathway has been suggested to contribute to evasion of immune surveillance by promoting the downregulation of cell-surface antigens that would be recognized by T-cells [242].

The MEK/ERK pathway is upstream of many of the other signaling nodes identified in our analysis, which indicates that it may be a master regulator of the majority of the signaling pathways and cascades involved in the pathogenesis of endometriosis and, therefore, highly important as a potential therapeutic target [241].

#### Prospective biomarkers

A great deal of research has recently been devoted to the identification of biomarkers for diagnosis of endometriosis. While some putative candidates have been identified, they lack sufficient sensitivity and specificity to be clinically useful. The results of our bioinformatics analysis align with those previous findings in that the pathways identified are ubiquitous key regulatory pathways. As such, they are involved in numerous signaling cascades controlling cell proliferation, apoptosis, invasion, and have implications in a variety of disease processes.

We used Metacore software for bioinformatics analysis of the main signaling pathways involved in pathogenesis of endometriosis. Metacore software provides an opportunity to model disease pathways and targets allow assessment of biomarkers. Of the pathways identified, perhaps the most viable as a biomarker are the STATs. A meta-analysis of biomarker studies identified numerous cytokines as being potentially diagnostic for endometriosis [172]. Many of these cytokines transduce signals through STATs. Therefore, STATs may serve as a convergent, and thereby more sensitive, readout of cytokine status.

#### Prospective treatment methods

In addition to their potential role as biomarkers of endometriosis the signaling molecules and pathways identified by our analysis may also provide insights into new therapies.

**JAK/STAT** The JAK/STAT pathway is a tempting target for novel therapeutics because it is relatively simple mechanistically, providing a direct translation of extracellular signal into a transcriptional response. There are several small molecule drug candidates that have been shown to regulate the JAK/STAT pathway and several are already on the market while others are still in clinical trials [244].

**Figure 7:**
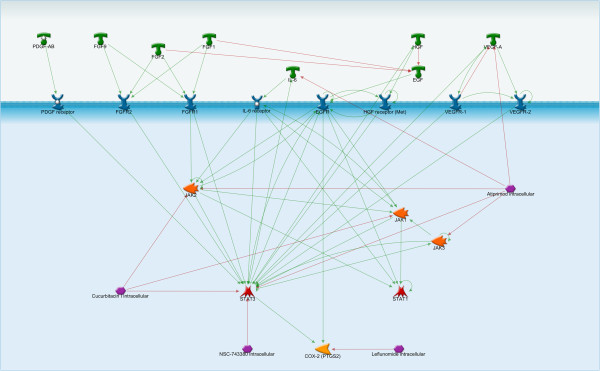
**JAK/STAT signaling pathway in endometriosis.** The effects of PDGF, FGF, IL-6, HGF, and VEGF are all primarily achieved through STAT activation. The JAK/STAT pathway is a tempting target for novel therapeutics because it is relatively simple mechanistically, providing a direct translation of extracellular signals into a transcriptional response. Of the pathways identified, perhaps the most viable as a biomarker are the STATs. Among the small molecule inhibitors of this pathway are Leflunomide (a JAK inhibitor) and Atiprimod (a STAT3 inhibitor).

Leflunomide is a JAK inhibitor that is used to treat rheumatoid arthritis (RA) and fibrosis. In model cell lines, leflunomide primarily inhibits cell migration through inhibitory effects on ECM proteins such as collagen [244]. Though it has also been shown to block cell proliferation through various JAK mediated pathways [Figure 7].

Atiprimod is a STAT3 inhibitor currently in clinical trials. In multiple myeloma cells lines it has been shown to induce apoptosis via cleavage of caspase-3 and downregulation of Bcl-2 [244]. Furthermore, it has been shown to have anti-inflammatory effects in animal models of RA via inhibition of IL-6 production [244].

**TGF-β/SMAD** Novel therapeutics aimed at inhibition of the TGF-β/SMAD pathway have taken three forms: (1) translational inhibition using antisense oligonucleotides, (2) inhibition of the ligand/receptor interaction using monoclonal antibodies, and (3) small molecule inhibitors of the signaling cascade [245]. Several antisense oligonucleotide therapies are in clinical trials and have demonstrated great efficacy in cell models. However, thus far, clinical results have been disappointing [245]. Alternatively, several monoclonal antibodies have shown significant promise.

**Figure 8:**
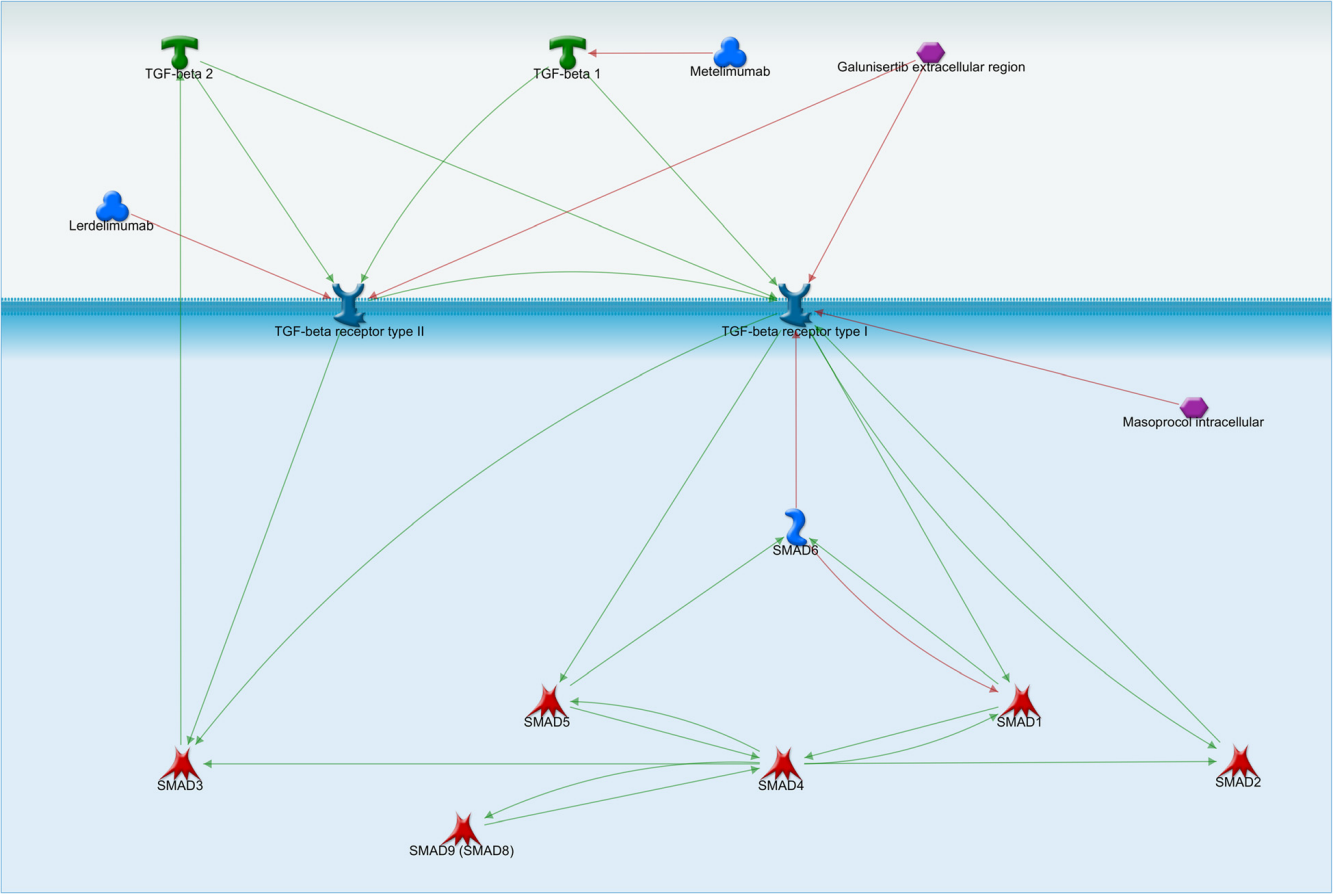
**TGF-β/SMAD signaling pathway.** The SMAD proteins are the only family of transcription factors known to propagate TGF-β signals. Lerdelimumab (a recombinant human IgG4 targeting TGF- β2) and Metelimumab (a human monoclonal IgG4 against TGF- β1) can inhibit TGF-β signaling.

Lerdelimumab is a recombinant human IgG4 targeting TGF-β2, which is currently in clinical trials for treatment of post-surgical fibrosis [245]. Metelimumab is a human monoclonal IgG4 against TGF-β1 currently being developed to treat scleroderma [245]. GC-1008 targets all TGF-β isoforms and is in clinical trials for treatment of melanoma and renal cell carcinoma [245]. Each of these antibodies showed enough promise in early stage clinical trials to warrant advancement to stage II/III clinical trials, which are still currently underway [Figure 8].

Several small molecule inhibitors are also being developed. SD-093 and LY-580276, which are primarily inhibitors of SMAD2/3 activity, have been shown to inhibit cell migration and invasion in cell culture models and are proceeding to clinical trials [245].

The TGF-β/SMAD signaling pathway is much more complex than that of JAK/STAT and is known to have dual roles in regulation of cell proliferation. The balance of pro-proliferative and anti-proliferative effects must be maintained in an effective therapy targeting this pathway. Therefore, therapeutic development will be complex.

**MEK/ERK** Perhaps the most well studied pathway, MEK/ERK signaling has several distinct advantages as a therapeutic target. Firstly, most MEK inhibitors that have been developed are very specific and do not inhibit many different protein kinases [241]. Secondly, unlike the TGF-β/SMAD pathway, targeting of MEK is highly specific as ERK is the only known effector and the downstream targets are fairly well defined [241]. Lastly, MEK/ERK signaling represents a convergence of many upstream signaling pathways that could be inhibited with a single therapeutic.

**Figure 9:**
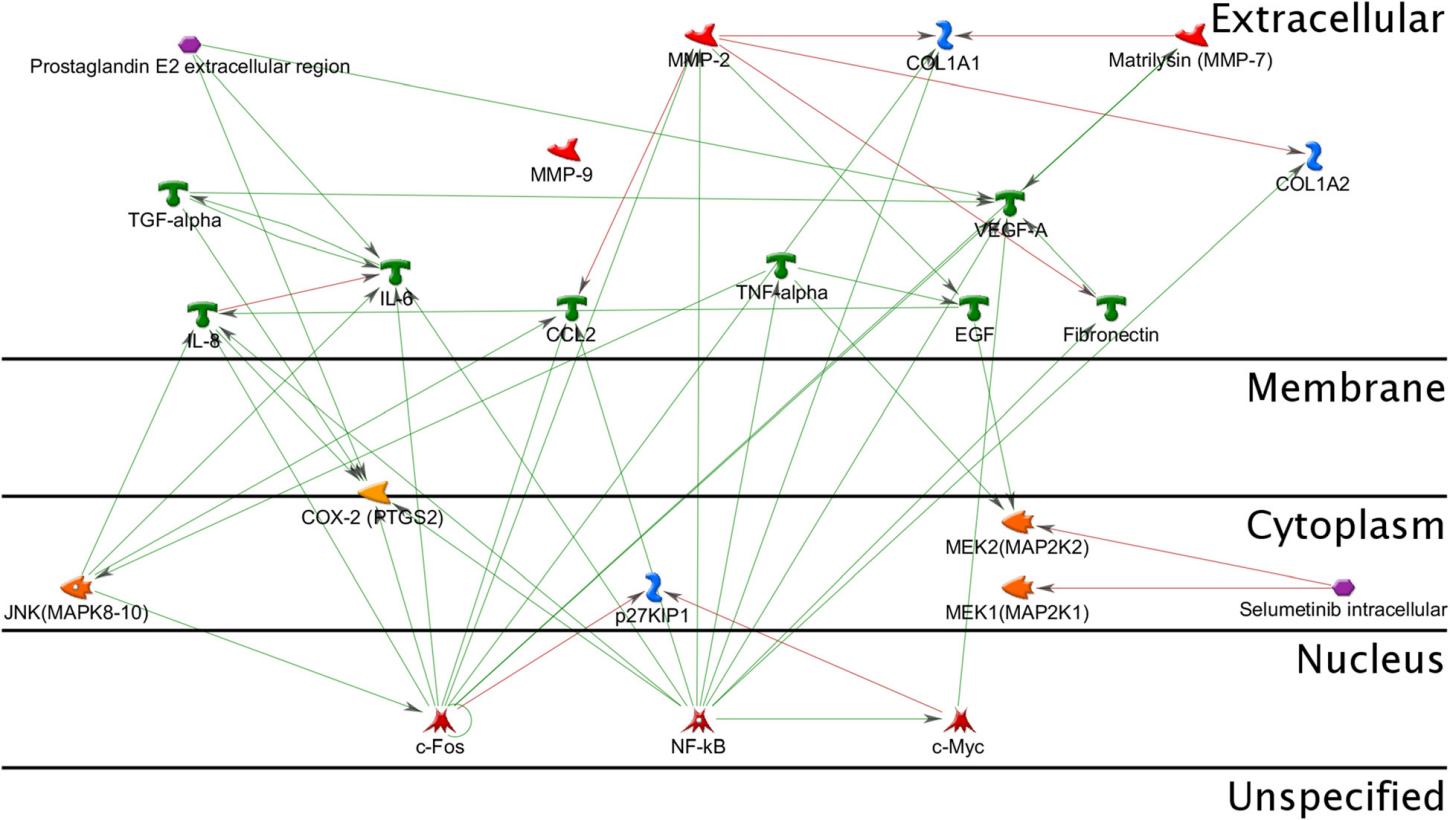
**Interactions between MEK, c-Myc, c-Fos and growth factors.** The effects of EGF and TNF-alpha are mediate via the MEK-pathway. Selumetinib and other MEK/ERK inhibitors are being primarily developed and studied for cancer treatment. However, the role of the MEK/ERK-pathway in endometriosis suggests that these therapeutic strategies may lend themselves equally well to this disease.

Selumetinib is a small molecular inhibitor of MEK that recently entered phase III clinical trials for treatment of non-small cell lung cancer. Selumetinib has been shown to be effective for inhibition of cell proliferation in several tumor models, but does not affect the growth of normal human cells [241]. It is thought that selumetinib is cytostatic, meaning that it inhibits proliferation via cell cycle arrest but does not induce apoptosis [Figure 9].

Selumetinib and other MEK/ERK inhibitors are being primarily developed and studied for cancer treatment. However, our analysis revealing the critical role of this particular pathway in endometriosis suggests that these therapeutic strategies may lend themselves equally well to this disease.

## Conclusions

Endometriosis is a common and painful condition affecting women of reproductive age and the prevalence is rapidly increasing internationally. However, the global government funding for and the number of research projects focusing on endometriosis have remained at the relatively same level since 2008 and represent a minor fraction of the female genitourinary cancers [178]. There needs to be the increased international effort to understand the etymology of endometriosis, explore preventative methods, and personalize treatment options.

While the underlying pathophysiology is still largely unknown, many advancements have been made in understanding the progression of the disease. In recent years, a great deal of research has focused on non-invasive diagnostic tools, such as biomarkers, as well as identification of potential therapeutic targets. The current diagnostic tools are invasive and current therapies primarily treat the symptoms of endometriosis. Optimally, the advancement of genomic and proteomic data will facilitate the development of non-invasive diagnostic biomarkers as well as therapeutics that target the pathophysiology of the disease and halt, or even reverse, progression. However, the amount of data generated by these types of studies is vast and bioinformatics analysis, such as we present here, will be critical to identification of appropriate targets for further study.

Our network analysis identified the STAT proteins, the SMAD transcription factors, and the MEK/ERK pathways as central regulators for the pathophysiological processes that drive development of endometriosis. Therefore, these proteins and pathways have the potential to yield clinically useful biomarkers and treatments for endometriosis.

## Competing interests

The authors declare that they have no competing interests.

## Authors’ contributions

YB has made substantial contributions to acquisition and analysis of data and has participated in the bioinformatics analysis. MB has been involved in drafting the manuscript. TK has participated in the review writing and has revised the draft critically. AM carried out the bioinformatics analysis. AA has revised the draft and has given final approval of the version to be published. All authors read and approved the final manuscript.
